# 2017 update of the WSES guidelines for emergency repair of complicated abdominal wall hernias

**DOI:** 10.1186/s13017-017-0149-y

**Published:** 2017-08-07

**Authors:** Arianna Birindelli, Massimo Sartelli, Salomone Di Saverio, Federico Coccolini, Luca Ansaloni, Gabrielle H. van Ramshorst, Giampiero Campanelli, Vladimir Khokha, Ernest E. Moore, Andrew Peitzman, George Velmahos, Frederick Alan Moore, Ari Leppaniemi, Clay Cothren Burlew, Walter L. Biffl, Kaoru Koike, Yoram Kluger, Gustavo P. Fraga, Carlos A. Ordonez, Matteo Novello, Ferdinando Agresta, Boris Sakakushev, Igor Gerych, Imtiaz Wani, Michael D. Kelly, Carlos Augusto Gomes, Mario Paulo Faro, Antonio Tarasconi, Zaza Demetrashvili, Jae Gil Lee, Nereo Vettoretto, Gianluca Guercioni, Roberto Persiani, Cristian Tranà, Yunfeng Cui, Kenneth Y. Y. Kok, Wagih M. Ghnnam, Ashraf El-Sayed Abbas, Norio Sato, Sanjay Marwah, Muthukumaran Rangarajan, Offir Ben-Ishay, Abdul Rashid K Adesunkanmi, Helmut Alfredo Segovia Lohse, Jakub Kenig, Stefano Mandalà, Raul Coimbra, Aneel Bhangu, Nigel Suggett, Antonio Biondi, Nazario Portolani, Gianluca Baiocchi, Andrew W Kirkpatrick, Rodolfo Scibé, Michael Sugrue, Osvaldo Chiara, Fausto Catena

**Affiliations:** 1 Department of Surgery, University of Bologna, Bologna, Italy; 2 Department of Surgery, Macerata Hospital, Macerata, Italy; 3 Department of Surgery, Maggiore Hospital, Bologna, Italy; 4 Department of General Surgery, Papa Giovanni XXIII Hospital, Bergamo, Italy; 5 Department of Surgery, Red Cross Hospital Beverwijk, Erasmus University Medical Center, Rotterdam, Netherlands; 6 Department of Surgical Science, Istituto Clinico Sant’Ambrogio, Milan, Italy; 7 Department of General Surgery, Mozyr City Hospital, Mazyr, Belarus; 8 Denver Health, University of Colorado, Denver, CO USA; 9 Department of Surgery, University of Pittsburgh School of Medicine, Pittsburgh, USA; 10 Division of Trauma, Emergency Surgery and Surgical Critical Care, Massachusetts General Hospital, Harvard Medical School, Boston, MA USA; 11 Department of Surgery, University of Florida, Gainesville, FL USA; 12 Department of Abdominal Surgery, University Hospital Meilahti, Helsinki, Finland; 13 Department of Surgery, University of Hawaii, Honolulu, HI USA; 14 Department of Primary Care and Emergency Medicine, Kyoto University Graduate School of Medicine, Kyoto, Japan; 15 Division of General Surgery, Rambam Health Care Campus, Haifa, Israel; 16 Division of Trauma Surgery, Hospital de Clinicas, School of Medical Sciences, University of Campinas, Campinas, Brazil; 17 Department of Surgery, Universidad del Valle, Fundacion Valle del Lili, Cali, Colombia; 18 General Surgery Clinic, Azienda ULSS 5 “Polesana”, Adria, RO Italy; 19 General Surgery Clinic, University Hospital St. George/Medical University of Plovdiv, Plovdiv, Bulgaria; 20 Department of Surgery 1, Lviv Regional Hospital, Danylo Halytsky Lviv National Medical University, Lviv, Ukraine; 21 Department of Surgery, Sheri-Kashmir Institute of Medical Sciences, Srinagar, India; 22 Griffith Base Hospital, Griffith, NSW Australia; 23 Federal University of Juiz de Fora (UFJF), Juiz de Fora, MG Brazil; 24 Faculdade de Ciências Médicas e da Saúde de Juiz de Fora (SUPREMA), Juiz de Fora, MG Brazil; 25 Department of General Surgery, Trauma and Emergency Surgery Division, ABC Medical School, Santo André, SP Brazil; 26 Department of Emergency Surgery, Maggiore Parma Hospital, Parma, Italy; 27 Department of Surgery, Tbilisi State Medical University, Tbilisi, Georgia; 28 Department of Surgery, Yonsei University College of Medicine, Seoul, South Korea; 29 Department of Surgery, Montichiari Hospital, ASST Spedali Civili Brescia, Brescia, Italy; 30 Department of Surgery, Mazzoni Hospital, Ascoli Piceno, Italy; 31 Catholic University of the Sacred Heart, Rome, Italy; 32 Department of Surgery, Tianjin Nankai Hospital, Nankai Clinical School of Medicine, Tianjin Medical University, Tianjin, China; 33 Department of Surgery, RIPAS Hospital, Bandar Seri Begawan, Brunei; 34 Department of Surgery Mansoura, Faculty of Medicine, Mansoura University, Mansoura, Egypt; 35 Department of Surgery, Pt. BDS Post Graduate Institute of Medical Sciences, Rohtak, India; 36 Department of Laparoscopic and Bariatric Surgery, Health City Cayman Islands, Grand Cayman, Cayman Islands; 37 Department of Surgery, College of Health Sciences, Obafemi Awolowo University Hospital, Ile-Ife, Nigeria; 38 II Cátedra de Clínica Quirúrgica, Hospital de Clínicas, Facultad de Ciencias Médicas, Universidad Nacional de Asunción, San Lorenzo, Paraguay; 39 3rd Department of General Surgery, Jagiellonian University Collegium Medium, Krakow, Poland; 40 Department of Surgery, G. Giglio Hospital Cefalù, Palermo, Italy; 41 Department of Surgery, Division of Trauma, Surgical Care, Burns and Acute Care Surgery, UC San Diego Medical Center, San Diego, CA USA; 42 Academic Department of Surgery, University Hospitals Birmingham NHS Foundation Trust, Edgabaston, Birmingham, UK; 43 Department of Colorectal Surgery, New Queen Elizabeth Hospital Birmingham, University Hospitals Birmingham NHS Foundation Trust, Edgbaston, Birmingham, UK; 44 University of Catania, Catania, Italy; 45 Department of Surgery, Brescia Hospital, Brescia, Italy; 46 Departments of Critical Care Medicine and Surgery, Foothills Medical Centre, Calgary, AB Canada; 47 Letterkenny Hospital, Donegal, Ireland; 48 Niguarda Hospital, Milan, Italy

**Keywords:** Hernia repair, Emergency surgery, Incarcerated hernia, Strangulated hernia, Mesh repair, Biologic mesh, Bowel resection, Infected field, Contaminated wound, Abdominal wall hernia

## Abstract

Emergency repair of complicated abdominal wall hernias may be associated with worsen outcome and a significant rate of postoperative complications. There is no consensus on management of complicated abdominal hernias. The main matter of debate is about the use of mesh in case of intestinal resection and the type of mesh to be used. Wound infection is the most common complication encountered and represents an immense burden especially in the presence of a mesh. The recurrence rate is an important topic that influences the final outcome. A World Society of Emergency Surgery (WSES) Consensus Conference was held in Bergamo in July 2013 with the aim to define recommendations for emergency repair of abdominal wall hernias in adults. This document represents the executive summary of the consensus conference approved by a WSES expert panel. In 2016, the guidelines have been revised and updated according to the most recent available literature.

## Background

A large number of abdominal hernias require emergency surgery. However, these procedures may be associated with poor prognosis and a significant rate of postoperative complications [1].

Abdominal hernias may be classified as groin hernias (femoral or inguinal) and ventral hernias (umbilical, epigastric, Spigelian, lumbar, and incisional).

An incarcerated hernia is a hernia in which the content has become irreducible due to a narrow opening in the abdominal wall or due to adhesions between the content and the hernia sac. Moreover, intestinal obstruction may complicate an incarcerated hernia. A strangulated hernia occurs when the blood supply to the contents of the hernia (e.g. omentum, bowel) is compromised [2]. Strangulated hernias remain a significant challenge, as they are sometimes difficult to diagnose by physical examination and require urgent surgical intervention. Early surgical intervention of a strangulated hernia with obstruction is crucial as delayed diagnosis can result in the need for bowel resection with prolonged recovery and increased complication rate. Strangulated hernias may lead to bacterial translocation and intestinal wall necrosis (potentially resulting in bowel perforation). This condition significantly increases the risks in emergency hernia repair that may lead to an increased incidence of surgical site contamination and recurrence.

An interesting topic is the use of laparoscopy in emergency hernia repair. However, its role in acute settings is not well established yet.

Bacteria inherently colonize all surgical wounds, but not all of these contaminations ultimately lead to infection. In most patients, infection does not occur because innate host defences are able to eliminate microbes at the surgical site. However, there is some evidence that the implantation of foreign materials, such as prosthetic mesh, may lead to a decreased threshold for infection [3].

While many factors can influence surgical wound healing and postoperative infection, bacterial burden is the most significant risk factor. According to the likelihood and degree of wound contamination at the time of operation, the Centers for Disease Control and Prevention (CDC) wound classification stratifies the wound as follows [4]:

- Class I = clean wounds
- Class II = clean-contaminated wounds
- Class III = contaminated wounds
- Class IV = dirty or infected wounds (Table 1)

**Table 1:** Surgical wound classification [4]

Class I/clean	An uninfected operative wound in which no inflammation is encountered and the respiratory, alimentary, genital, or uninfected urinary tract is not entered. In addition, clean wounds are primarily closed and, if necessary, drained with closed drainage. Operative incisional wounds that follow non-penetrating (blunt) trauma should be included in this category if they meet the criteria
Class II/clean-contaminated	An operative wound in which the respiratory, alimentary, genital, or urinary tract is entered under controlled conditions and without unusual contamination. Specifically, operations involving the biliary tract, appendix, vagina, and oropharynx are included in this category, provided no evidence of infection or major break in technique is encountered
Class III/contaminated	Open, fresh, accidental wounds. In addition, operations with major breaks in sterile technique (e.g. open cardiac massage) or gross spillage from the gastrointestinal tract, and incisions in which acute, non-purulent inflammation is encountered are included in this category
Class IV/dirty-infected	Old traumatic wounds with retained devitalized tissue and those that involve existing clinical infection or perforated viscera. This definition suggests that the organisms causing postoperative infection were present in the operative field before the operation

The choice of technique repair is based on the contamination of the surgical field, the size of the hernia, and the experience of the surgeon.

In clean-contaminated, contaminated, and dirty surgical procedures, the polymicrobial aerobic and anaerobic flora closely resemble the normal endogenous microflora of the gastrointestinal (GI) tract and are the most frequently observed pathogens. The contaminating pathogens in GI surgery include gram-negative bacilli (e.g. *Escherichia coli*) and gram-positive microbes, such as enterococci and anaerobic organisms. A classification scheme has been demonstrated in multiple studies to predict the relative probability that a given wound will become infected [5, 6].

Several studies show clear advantages of mesh use in elective cases, where infection is uncommon [7]. Mesh is easy to use, has low complication rates, and significantly reduces the rate of hernia recurrence. However, few studies have investigated the outcome of mesh use in an emergency setting, where there is often surgical field contamination due to bowel involvement [8, 9].

The use of biological mesh has many advantages, including a decreased immune response, as well as decreased incidence of fistulae formation, fibrosis, and erosions.

There is, however, a paucity of high-quality evidence on the superiority of biological mesh, and it is still a very expensive device [10].

The role of local anaesthesia in the treatment of complicated inguinal and femoral hernia needs to be taken into consideration because of its multiple advantages, especially in patients with multiple comorbidities.

A World Society of Emergency Surgery (WSES) Consensus Conference was held in Bergamo in July 2013, during the 2nd Congress of the World Society of Emergency Surgery with the goal of defining recommendations for emergency repair of abdominal wall hernias in adults. This document represents the executive summary of the consensus conference approved by a WSES expert panel. In 2017, the guidelines have been revised and updated according to the most recent available literature (Appendix).

### Materials and methods

A computerized search was done by the bibliographer in different databanks (MEDLINE, Scopus, Embase), and citations were included for the period between January 2000 and December 2016 using the primary search strategy: hernia, groin, inguinal, femoral, crural, umbilical, epigastric, spigelian, ventral, incisional, incarcerated, strangulated, acute, emergency, repair, suture, mesh, direct, synthetic, polypropylene, prosthetic, biologic, SSI, wound infection, bowel resection, intestinal resection, complication, morbidity, recurrence, timing, laparoscopy combined with AND/OR. No search restrictions were imposed. The dates were selected to allow comprehensive published abstracts of clinical trials, consensus conference, comparative studies, congresses, guidelines, government publication, multicenter studies, systematic reviews, meta-analysis, large case series, original articles, and randomized controlled trials. Narrative review articles were also analysed to determine other possible studies. Recommendation guidelines are evaluated according to the Grading of Recommendations, Assessment, Development and Evaluation (GRADE), a hierarchical, evidence-based rubric [11, 12] summarized in Table 2.

**Table 2:** Grading of Recommendations, Assessment, Development and Evaluation (GRADE) from Guyatt and colleagues and Brozek et al. [11, 12]

Grade of recommendation	Clarity of risk/benefit	Quality of supporting evidence	Implications
1A			
Strong recommendation, high-quality evidence	Benefits clearly outweigh risk and burdens, or vice versa	RCTs without important limitations or overwhelming evidence from observational studies	Strong recommendation, applies to most patients in most circumstances without reservation
1B			
Strong recommendation, moderate-quality evidence	Benefits clearly outweigh risk and burdens, or vice versa	RCTs with important limitations (inconsistent results, methodological flaws, indirect analyses, or imprecise conclusions) or exceptionally strong evidence from observational studies	Strong recommendation, applies to most patients in most circumstances without reservation
1C			
Strong recommendation, low-quality or very low-quality evidence	Benefits clearly outweigh risk and burdens, or vice versa	Observational studies or case series	Strong recommendation but subject to change when higher-quality evidence becomes available
2A			
Weak recommendation, high-quality evidence	Benefits closely balanced with risks and burden	RCTs without important limitations or overwhelming evidence from observational studies	Weak recommendation, the best action may differ depending on the patient, treatment circumstances, or social values
2B			
Weak recommendation, moderate-quality evidence	Benefits closely balanced with risks and burden	RCTs with important limitations (inconsistent results, methodological flaws, indirect or imprecise) or exceptionally strong evidence from observational studies	Weak recommendation, the best action may differ depending on the patient, treatment circumstances, or social values
2C			
Weak recommendation, low-quality or very low-quality evidence	Uncertainty in the estimates of benefits, risks, and burden; benefits, risk, and burden may be closely balanced	Observational studies or case series	Very weak recommendation; alternative treatments may be equally reasonable and merit consideration

*RCTs* randomized controlled trials

The guidelines statements have been issued to each class according to the CDC wound classification (Table 1).

In 2016, the guidelines have been revised and updated by the WSES working group on emergency repair of complicated abdominal wall hernias according to the most recent literature available.

## Recommendations

### Timing of intervention

Patients should undergo emergency hernia repair immediately when intestinal strangulation is suspected (grade 1C recommendation).

Systemic inflammatory response syndrome (SIRS), contrast-enhanced CT findings, as well as lactate, serum creatinine phosphokinase (CPK), and D-dimer levels are predictive of bowel strangulation (grade 1C recommendation).

Unfortunately, morbidity and mortality rates remain high for patients who undergo emergency repair of abdominal hernias. Early diagnosis of strangulated obstruction may be difficult, and delayed diagnosis can lead to septic complications. However, in the case of suspected bowel strangulation, the benefits outweigh the risks of surgery and patients should undergo immediate surgical intervention.

A recent study performed by Martínez-Serrano et al. prospectively analysed morbidity and mortality rates following emergency hernia repair. The study population included 244 patients with complicated abdominal wall hernias requiring surgical repair. In this study, the patients were treated according to standardized protocols with detailed actions taken during the pre-, intra-, and postoperative periods. Clinical outcomes were compared retrospectively to that of 402 patients who had undergone similar procedures before the development and implementation of the protocols outlined in the study. Results showed higher rates of mortality in patients with acute complication as their first hernia-related symptom and whose treatment was delayed for more than 24 h. Thus, the authors concluded that early detection of complicated abdominal hernias may be the best means of reducing the rate of mortality [13].

Similar results were achieved in the study published in 2014 by Koizumi et al., retrospectively analysing the clinical course and outcomes in 93 patients with strangulated inguinal end femoral hernias. The results demonstrated how the elapsed time from onset to surgery was the most important prognostic factor (*P* < 0.005) [14].

In 2007, Derici et al. published a retrospective study using univariate and multivariate analyses to investigate factors affecting morbidity and mortality rates in cases of incarcerated abdominal wall hernias [15]. Using the univariate analysis, results showed that symptomatic periods lasting longer than 8 h, the presence of comorbid disease, high American Society of Anesthesiologists (ASA) scores, the use of general anaesthesia, the presence of strangulation, and the presence of necrosis significantly affect morbidity rates. In contrast, advanced age, the presence of comorbid diseases, high ASA scores, the presence of strangulation, the presence of necrosis, and hernia repair with graft were found to significantly affect mortality rates by univariate analysis; the presence of necrosis, however, was the only factor that appeared to significantly affect mortality rates based on multivariate analysis [16].

A retrospective study evaluated the risk factors associated with bowel resection and treatment outcome in patients with incarcerated groin hernias. The study analysed 182 adult patients with incarcerated groin hernias who underwent emergency hernia repair in the 10-year period from January 1999 to June 2009. Of these patients, bowel resection was required in 15.4% of cases (28/182). A logistic regression model identified three independent risk factors for bowel resection: lack of health insurance (odds ratio (OR) = 5, *P* = 0.005), obvious peritonitis (OR = 11.52, *P* = 0.019), and femoral hernia (OR = 8.31, *P* < 0.001) [17].

Many authors reported that early detection of progression from an incarcerated hernia to a strangulated hernia is difficult to achieve by either clinical or laboratory means, which presents a large challenge in early diagnosis [18–20]. Signs of SIRS including fever, tachycardia, and leukocytosis, as well as abdominal wall rigidity, are considered common indicators of strangulated obstruction. However, an investigation by Sarr et al. demonstrated that the combination of four classic signs of strangulation—continuous abdominal pain, fever, tachycardia, and leukocytosis—could not distinguish strangulated from simple obstructions [18]. Furthermore, Shatlla et al. reported a low incidence of these classical findings and stated that their presence indicated an advanced stage of strangulation, which would be of limited value for early diagnosis [19]. In 2004, Tsumura et al. published a retrospective study investigating SIRS as a predictor of strangulated small bowel obstruction. Multivariate analysis revealed that the presence of SIRS alongside abdominal muscle guarding was independently predictive of strangulated small bowel obstruction [21].

Among possible diagnostic tests, CPK appears to be a relatively reliable indicator of early intestinal strangulation [22, 23]. Icoz et al. published a prospective study investigating the relevance of serum D-dimer measurement as a potential diagnostic indicator of strangulated intestinal hernia. The authors concluded that D-dimer assays should be performed on patients presenting with intestinal emergencies to better evaluate and predict ischemic events. Despite having low specificity, elevated D-dimer levels measured upon admission were found to correlate strongly with intestinal ischaemia [24].

In 2012, an interesting retrospective study examining whether various laboratory parameters could predict the viability of strangulation in patients with bowel obstruction was published. Forty patients diagnosed with bowel strangulation operated within 72 h of the start of symptoms were included in the study. Lactate level was the only laboratory parameter significantly associated with a lack of viability (*P* < 0.01, Mann–Whitney *U* test). Other laboratory data did not show statistically significant associations. The authors concluded that an arterial blood lactate level of 2.0 mmol/L or greater was a useful predictor of non-viable bowel strangulation [25].

Early diagnostic methods to detect bowel strangulation have advanced substantially following the development and refinement of radiological techniques, such as computed tomography (CT) scanning [26]. Jancelewicz et al. published a retrospective analysis demonstrating that CT findings of reduced wall enhancement were the most significant independent predictor of bowel strangulation, with 56% sensitivity and 94% specificity. By contrast, elevated white blood cell (WBC) count and guarding on physical examination were only moderately predictive. It should be noted, however, that an elevated WBC was the only variable found to be independently predictive of bowel strangulation in patients with small bowel obstruction [27].

In 2014, Kahramanca et al. retrospectively analysed the role of WBC count and fibrinogen as predictive factors of incarcerated abdominal hernia. Comparing 100 patients with incarcerated hernia with 100 patients with uncomplicated hernia, the results showed that high levels of WBC and fibrinogen were significantly predictive of morbidity and cost burden (*P* < 0.001) [28].

### Laparoscopic approach

Diagnostic laparoscopy may be a useful tool with the target of assessing bowel viability after spontaneous reduction of strangulated groin hernias (grade 2B recommendation).

Repair of incarcerated hernias—both ventral and groin—may be performed with a laparoscopic approach in the absence of strangulation and suspicion of the need of bowel resection, where an open pre-peritoneal approach is preferable (grade 2C recommendation).

Few studies have focused on the laparoscopic approach to hernia repair in an emergency setting.

In 2004, Landau and Kyzer published a retrospective study investigating the use of laparoscopy in the repair of incarcerated incisional and ventral hernias. The authors argued that laparoscopic repair was feasible and could be safely used to treat patients presenting with incarcerated incisional and ventral hernias [29].

In 2007, a series of patients with large irreducible groin hernias (omentoceles), treated by laparoscopy without conversions, was published. The authors described a technique to facilitate complete removal of the hernia contents. A laparoscopic transperitoneal repair for large irreducible scrotal hernias, removing as much omentum as possible, was performed. Then, a small groin incision was made to excise the adherent omentum from the distal sac [30].

Another retrospective study published in 2008 investigated the role of laparoscopy in the management of incarcerated (non-reducible) ventral hernias. The authors concluded that laparoscopic repair of ventral abdominal wall hernias could be safely performed with low subsequent complication rates, even in the event of an incarcerated hernia. Careful bowel reduction with adhesiolysis and mesh repair in an uncontaminated abdomen (without inadvertent enterotomy) using a 5-cm-mesh overlap was an important factor predictive of successful clinical outcome [31].

In 2009, a retrospective study investigating laparoscopic techniques used to treat incisional hernias in an emergency setting was published. The results of this series also demonstrated the feasibility of laparoscopic surgery to treat incarcerated incisional hernias in an emergency setting [32].

Additionally, a systematic literature review performed in 2009 identified articles reporting on laparoscopic treatment, reduction, and repair of incarcerated or strangulated inguinal hernias from 1989 to 2008. It included seven articles on this topic, reporting on 328 cases treated with total extraperitoneal (TEP) or transabdominal preperitoneal (TAPP) repair. Laparoscopy can also be used to resect bowel, if necessary, or to repair an occult contralateral hernia, present in 11.2–50% of cases. The authors concluded that the laparoscopic repair is a feasible procedure with acceptable results; however, its efficacy needs to be studied further, ideally with larger, multicentre randomized controlled trials [33].

The retrospective 4-year analysis of 188 patients who underwent emergency surgical repair of strangulated groin hernias (57 laparoscopic and 131 open, including one and ten bowel resections, respectively, *P* = 0.117) revealed a significant lower wound infection rate (*P* < 0.018) in the laparoscopic group, without a higher recurrence rate (*P* < 0.815) [34].

Hernioscopy is a mixed laparoscopic–open surgical technique for incarcerated inguinal hernias. Specifically, it is effective in evaluating the viability of the herniated loop, thus avoiding unnecessary laparotomy [35].

A prospective randomized study in 2009 aimed to evaluate the impact of hernia sac laparoscopy on the morbidity and mortality of cases with a spontaneous reduction of the strangulated hernia content before the assessment of its viability. Ninety-five patients were randomly assigned to two groups: group A (21 patients managed using hernia sac laparoscopy) and group B (20 patients managed without laparoscopy). The median hospital stay was 28 h for group A and 34 h for group B. Four patients of group B had major complications, whereas there was none observed in group A. Two unnecessary laparotomies and two deaths occurred in group B. The authors concluded that hernia sac laparoscopy seems to be an accurate and safe method of preventing unnecessary laparotomy, and in high-risk patients, it contributes to decreased morbidity [36]..

### Emergency hernia repair in “clean surgical field” (CDC wound class I)

The use of mesh in clean surgical fields (CDC wound class I) is associated with lower recurrence rate, if compared to tissue repair, without an increase in the wound infection rate. Prosthetic repair with a synthetic mesh is recommended for patients with intestinal incarceration and no signs of intestinal strangulation or concurrent bowel resection (clean surgical field) (grade 1A recommendation).

#### Ventral hernias

For patients with intestinal incarceration and no signs of intestinal strangulation or concurrent bowel resection, the surgical field is presumed clean and the infectious risk for synthetic mesh is low. The absence of intestinal wall ischaemia makes patients less prone to bacterial translocation.

Advantages have demonstrated using a mesh for hernia repair in clean fields; such advantages include low rate of long-term complications and reduction of recurrence [37–42].

A wide variety of small-sized retrospective studies comparing mesh use to suture repair in the treatment of acute irreducible hernias have been published [39, 43, 44]. The prospective randomized trial by Abdel-Baki et al. compared the use of mesh repair (group 1, 21 patients) and tissue repair (group 2, 21 patients) in 42 cases with acute para-umbilical hernia. The wound infection rate between the two groups was not statistically significant. At follow-up (mean 16 ± 5.5 months), there were four recurrences in group 2 (4/21, 19%) and no recurrences in group 1 (*P* < 0.05) [42].

The prospective 6-year study by Abd Ellatif et al. included 115 patients who underwent acutely incarcerated abdominal wall hernia repair. The results showed low rates of wound infection (4.3%) and recurrence (4.3%), with a mean follow-up of 42 months. The authors therefore concluded that mesh hernioplasty is crucial to prevent recurrence and that it is safe for repairing acutely incarcerated hernias [45].

#### Groin hernias

The retrospective study by Venara et al. compared the 30-day outcome after acute hernia (inguinal, femoral, and umbilical) repair with or without mesh. The study included 166 patients, of which 64 were treated with and 102 without mesh repair. Among the 64 patients who underwent mesh repair, four patients had concomitant bowel resection. Among the 102 patients who underwent primary repair, 21 patients had concomitant bowel resection. The mesh repair was neither related to a significant increase of complications (*P* = 0.89) nor related to surgical site infection (SSI) (*P* = 0.95), overall morbidity (OR = 1.5, confidence interval (CI) = 95%, *P* = 0.458), and major complications (OR = 1.2, CI = 95%, *P* = 0.77) [37].

A recent prospective study included 202 patients with acutely incarcerated groin hernias. The results showed extremely low rates of wound infection, mesh infections, and recurrence. The authors concluded that the use of mesh in incarcerated hernias is safe [46].

### Emergency hernia repair in “clean-contaminated surgical field” (CDC wound class II)

For patients having a complicated hernia with intestinal strangulation and/or concomitant need of bowel resection without gross enteric spillage (clean-contaminated surgical field, CDC wound class II), emergent prosthetic repair with a synthetic mesh can be performed (without any increase in 30-day wound-related morbidity) and is associated with a significant lower risk of recurrence, regardless the size of hernia defect (grade 1A recommendation).

The use of prosthetic grafts in clean-contaminated settings is seldom described. Most studies on the subject focus on elective repair.

#### Ventral hernias

In 2000, Mandalà et al. published a series of patients with incisional hernias treated with non-absorbable prostheses and associated visceral surgery. The low incidence of suppurative complications, with neither removal of the patch nor recurrences in the short term, showed that non-absorbable mesh repair in potentially contaminated fields was safe [47].

Retrospective studies by Vix et al., Birolini et al., and Geisler et al. report wound-related morbidity rates of 10.6, 20, and 7%, respectively, following mesh use in both clean-contaminated and contaminated procedures [48–50].

The retrospective study by Campanelli et al. analysed ten prosthetic hernia repairs in potentially contaminated fields and reported no major or minor complications after a 21-month follow-up period [51].

On the other hand, in 2010, Xourafas et al. retrospectively examined the impact of mesh use on ventral hernia repairs with simultaneous bowel resections attributable to either cancer or bowel occlusion. Researchers found a significantly higher incidence of postoperative infection in patients with a prosthetic mesh compared to those without mesh. According to the multivariate regression analysis, prosthetic mesh use was the only significant risk factor, irrespective of other variables such as drain use, defect size, or type of bowel resection [52].

The large-sized US National Surgical Quality Improvement Program (NSQIP) study by Choi et al., analysed and compared postoperative outcome following ventral hernia repair, in the 5-year period from 1 January 2005 to 4 April 2010, including 6721 clean-contaminated cases, of which 3879 underwent mesh repair and 2842 underwent non-mesh repair. The results did not show a significant statistical difference in the rate of deep incisional SSI and return to OR within 30 days, between the mesh and non-mesh groups [53].

One of the few available studies investigating acute hernia repair is the small-sized retrospective analysis by Nieuwenhuizen et al. including 23 patients who underwent acute hernia repair with intestinal resection, and surprisingly, it revealed a higher incidence of wound infection in the primary suture group (5/14, 35%) than in the mesh group (2/9, 22%) [54].

Another retrospective analysis of emergency prosthetic repair of incarcerated incisional hernias with simultaneous bowel resection in potentially contaminated fields including 60 patients demonstrated that the intestinal resection was associated with high rates of wound infection (38%) [55].

The prospective 6-year study by Abd Ellatif et al. included 163 patients who underwent acutely incarcerated abdominal wall hernia mesh repair, of which 48 required intestinal resection and anastomosis and 155 did not. No significant difference was found in terms of postoperative morbidities, wound infection, and recurrence rate between the two groups. The authors therefore concluded that mesh hernia repair is crucial to prevent recurrence and that it is safe for repairing acutely incarcerated hernias, even in case of intestinal resection [45].

In 2013, a prospective study to present a 7-year experience with the use of prosthetic mesh repair in the management of the acutely incarcerated and/or strangulated ventral hernias was published. Resection–anastomosis of non-viable small intestine was performed in 18 patients (23%) and was not regarded as a contraindication for prosthetic repair [43].

Haskins et al. evaluated the outcomes after emergency ventral hernia repair in 1357 patients with CDC wound class II from the American College of Surgeons (ACS) NSQIP database and did not find any statistical significance in wound-related or additional 30-day patient morbidity or mortality, between mesh and non-mesh emergency ventral hernia repair. The authors concluded that emergency ventral hernia repair with a mesh can be safely performed without an increase in wound-related or additional early patient morbidity or mortality in CDC wound class II [56].

The randomized trial by Kassem and El-Haddad compared the use of onlay polypropylene mesh positioned and supported by omentum and/or peritoneum versus inlay implantation of polypropylene-based composite mesh in 60 patients with complicated wide-defect ventral hernias, including 12 bowel resections. Postoperatively, seven patients developed a wound infection (11.6%) and two patients developed a recurrence (3%), after 3 and 8 months, respectively [57].

#### Groin hernias

Some studies have asserted that prosthetic repair of abdominal hernias can be safely performed alongside simultaneous colonic operations. Such joint procedures, they argue, exhibit acceptable rates of infectious complications and recurrence, and consequently, they stated that there is insufficient evidence to advocate the avoidance of prosthetic mesh in clean-contaminated fields, assuming that the appropriate technique is used [44, 58].

Also, the results of the retrospective study by Ueda et al. including 27 patients operated for strangulated groin hernia with small bowel resection (ten patients with mesh and 17 without mesh) did not show any statistically significant differences in terms of morbidity between the two groups and led to the conclusion that strangulated inguinal hernia cannot be considered a contraindication to the mesh repair even in case of intestinal resection [59].

A recent prospective study by Bessa et al. enrolled 234 patients with acutely or strangulated groin hernias of which 34 underwent resection and anastomosis of non-viable intestine. The results did not show any significant difference (*P* = 0.7) in the rate of wound or mesh infection between hernias with viable versus non-viable contents. The authors concluded that the presence of non-viable intestine could not be regarded as a contraindication for prosthetic repair [46].

In the retrospective study by Venara et al. including a subgroup of 25 patients who underwent acute hernia repair with concomitant bowel resection (four with mesh repair and 21 with primary repair), bowel resection appeared to be a risk factor for overall postoperative complications (*P* > 0.0001) and major complications (*P* = 0.003), but not for postoperative SSI (*P* = 0.42). The authors concluded that mesh repair appeared to be safe in the treatment of incarcerated hernia, since after multivariate analysis, mesh placement was not a significant predictor of postoperative complication (*P* = 0.458) [37].

In 2014, a SR and meta-analysis including nine studies has been published, investigating the optimal technique to treat strangulated inguinal hernia (mesh vs non-mesh repair). The wound infection rate has been found to be lower in the mesh group than in the control group (OR = 0.46, CI = 95%, *P* = 0.07). The recurrence rate was found to be lower in the mesh repair group (OR = 0.2, CI = 95%, *P* = 0.02). Nonetheless, the authors concluded that the study did not allow to currently recommend the use of mesh in case of bowel resection, despite the finding of similar SSI rates with either mesh repair or non-mesh techniques, when comparing bowel resection and no bowel resection (OR = 1.50, *P* = 0.73) [60].

### Emergency hernia repair in “contaminated-dirty surgical field” (CDC wound classes III and IV)

For stable patients with strangulated hernia with bowel necrosis and/or gross enteric spillage during intestinal resection (contaminated, CDC wound class III) or peritonitis from bowel perforation (dirty surgical field, CDC wound class IV), primary repair is recommended when the size of the defect is small (< 3 cm); when direct suture is not feasible, a biological mesh may be used for repair (grade 2C recommendation).

The choice between a cross-linked and a non-cross-linked biological mesh should be evaluated depending on the defect size and degree of contamination (grade 2C recommendation).

If a biological mesh is not available, either polyglactin mesh repair or open wound management with delayed repair may be a viable alternative (grade 2C recommendation).

In cases of bacterial peritonitis, patients must undergo contaminated surgical intervention, which means that the surgical field is infected and the risk of surgical site infection is very high.

High infection rates are reported after emergency hernia repairs with a polypropylene mesh of CDC wound class III. A retrospective study by Kelly and Behrman reported a 21% infection rate in a series of emergency and elective incisional hernia repairs [61]. Recently, a retrospective study by Carbonell et al. investigated open ventral hernia repairs performed with a polypropylene mesh in the retro-rectus position in clean-contaminated and contaminated fields: the 30-day surgical site infection rate was 7.1 and 19.0%, respectively [62].

Some authors investigated the use of absorbable prosthetic materials [64]. However, the use of absorbable prosthesis exposes the patient to an inevitable hernia recurrence. These meshes, once implanted, induce an inflammatory reaction that, through a hydrolytic reaction, digests and removes and digests the implanted prosthetic material completely. In this case, the high risk of hernia recurrence is explained by the complete dissolution of the prosthetic support [63].

Biological mesh prosthetics are most commonly used in infected fields involving large, complex abdominal wall hernia repairs. The use of biological mesh, which becomes vascularized and remodelled into autologous tissue after implantation, may offer a low-morbidity alternative to prosthetic mesh products in these complex settings, with good results also in immune-compromised patients [64]. By incorporating a biological mesh, surgeons hope to provide a collagen-based extracellular matrix scaffold by which host fibroblasts can induce angiogenesis and deposit new collagen. The non-synthetic material of biological mesh makes it less susceptible to infection, and several biological grafts are available in the current market. The classification of biological meshes is based on the species of origin (allogenic or xenogenic), the type of collagen matrix utilized (dermis, pericardium, or intestinal submucosa), the decellularization process, the presence or absence of cross-linkage, temperature-related storage requirements, and the use of rehydration [65]. On the basis of either the presence or not of the cross-linking, biological prostheses are divided into two subgroups: the partially remodelling ones (cross-linked) and the completely remodelling ones (not cross-linked). Thanks to the presence of additional links, the partially remodelling ones resist better and for a longer period to mechanical stress [64].

Many retrospective studies have explored the promising role of biological mesh in contaminated fields, but most of these investigations did not focus on emergency repair of incarcerated hernias [66–86]. Although a biological mesh in these situations is safe, long-term durability has still not been demonstrated [87–89].

A recent multicentre large-sized retrospective study compared suture, synthetic mesh, and biologic matrix in contaminated ventral hernia repair. On multivariate analysis, a biologic matrix was associated with a non-significant reduction in both SSI and recurrences, whereas a synthetic mesh was associated with fewer recurrences compares to suture and non-significant increase in SSI [90].

A prospective study by Catena et al. published in 2007 focused on complicated incisional hernia repair using mesh prosthetics made of porcine dermal collagen (PDC). Incisional hernioplasty using PDC grafts was found to be a safe and efficient approach to difficult contaminated cases [81].

Coccolini et al. published the results of the first 193 patients of the Italian Register of Biological Prosthesis (IRBP) [86]. This prospective multicentre study suggests the usefulness, versatility, and ease of using biological prosthesis in many different situations, including contaminated surgical fields.

The literature review by Coccolini et al. covered the use of biological meshes for abdominal reconstruction in emergency and elective setting in transplanted patients and reported a complication rate of 9.4% [84].

In 2014, Han et al. published a retrospective study including 63 patients who underwent emergency surgery for acute incarcerated abdominal wall hernias with human acellular dermal matrix (ADM) repair with a very low rate of infection (1.6%) as well as recurrences (15.9%) in a follow-up of 43 months. Bowel resection, performed in 33 patients, did not significantly affect the bulge and recurrence rate (*P* = 0.262). Interestingly, multivariate analysis demonstrated three factors to be significantly related to bulge and recurrence: BMI (*P* = 0.008), defect size (*P* = 0.016), and numbers of biological meshes used (*P* = 0.027) [91].

The systematic review by Lee et al. included a total of 32 studies regarding the use of synthetic and biologic materials for abdominal wall reinforcement in contaminated fields. In contaminated and/or dirty fields, wound infection rates were similar, but pooled hernia rates were 27.2% (95% CI = 9.5–44.9) with biological and 3.2% (95% CI = 0.0–11.0) with synthetic non-absorbable meshes. Other outcomes were comparable [92].

The recent multicentre prospective observational study by De Simone et al. included 71 patients who underwent emergency ventral hernia repair with a biological mesh. The surgical field resulted contaminated in 27 patients (38%), potentially contaminated in 19 patients (26.7%), and dirty in 25 patients (35.2%). Early postoperative (3rd–7th postoperative days) wound infection occurred in 21 patients (29.57%). High ASA score (≥ 3) (OR = 2.82, CI = 1.85–6.43, *P* = 0.03), smoking (OR = 4.1, CI = 1.73–6.35, *P* = 0.02), diabetes (OR = 3.23, CI = 1.92–4.38, *P* = 0.04), chronic immunosuppression (OR = 2.41, CI = 0.33–5.25, *P* = 0.003), previous hernia repair (OR = 1.99, CI = 1.5–2.9, *P* = 0.002), dirty surgical field (OR = 1.87, CI = 0.35–4.4, *P* = 0.04), sublay extraperitoneal bio-prosthesis placement (OR = 0.45, CI = 0.27–1.13, *P* = 0.009), and no anterior fascia closure (OR = 0.33, CI = 0.2–2.3, *P* = 0.04) were associated with wound complications. After a mean follow-up time of 27.2 months, hernia recurrence occurred in 19 patients (26.76%) [93].

Haskins et al. evaluated the outcomes after emergency ventral hernia repair in 1092 patients from the ACS NSQIP database and did not find any statistical significance in wound-related or additional 30-day patient morbidity or mortality, between mesh and non-mesh emergency ventral hernia repair. The authors concluded that emergency ventral hernia repair with a mesh can be safely performed without an increase in early wound-related or additional 30-day patient morbidity or mortality in CDC wound classes III and IV [56].

The use of biological materials in clinical practice has led to innovative methods of treating abdominal wall defects in contaminated surgical fields, although there is still an insufficient level of high-quality evidence on their value, and there is still a very huge price difference between the synthetic and biological meshes [10]. All literature reviews found in the MEDLINE database supported biologic mesh use in the setting of contaminated fields, but the literature included in these reviews consisted of case series and case reports with low levels of evidence [94]. Despite the lack of a cohesive body of evidence, published studies on biological mesh suggest that cross-linked mesh prosthetics have the lowest failure rate in contaminated and outright infected fields. To better guide surgeons, prospective randomized trials should be undertaken to evaluate the short- and long-term outcomes associated with biological meshes [90, 95].

For unstable patients (experiencing severe sepsis or septic shock), open management is recommended to prevent abdominal compartment syndrome; intra-abdominal pressure may be measured intraoperatively (grade 2C recommendation).

A prospective study published by Beltrán et al. examined 81 consecutively unselected patients presenting with complicated hernias and intestinal obstruction. The researchers used intra-abdominal pressure, measured with the intravesicular pressure method, to assess the clinical severity of strangulated hernias and predict intestinal strangulation [96]. Patients with intestinal strangulation and peritonitis are critically ill cases, commonly shocked and at high risk of septic complications; these patients may experience high intraoperative intra-abdominal pressure. Such hypertension may be the underlying cause of increased pulmonary pressures, reduced cardiac output, splanchnic hypoperfusion, and oliguria, leading to an abdominal compartment syndrome. Increased pressure within the constricting abdominal compartment in conjunction with unchanging or more likely disease-induced reduced abdominal compliance will also greatly reduce visceral perfusion within the abdominal compartment leading to an acute bowel injury [97–99]. This “acute bowel injury” results in release of pro-inflammatory mediators into the peritoneum and systemic circulation, leading to neutrophil priming, increased intestinal wall permeability, extravasation of fluid into the bowel wall and mesentery, translocation of intestinal bacteria, and absorption of bacterial endotoxin [100–103]. Even relatively mild intra-abdominal hypertension (IAH) (e.g. an IAP of 15 mmHg) has been reported to decrease intestinal microcirculatory blood flow, increase bowel wall permeability, and induce irreversible gut histopathological changes, bacterial translocation, and multi-organ dysfunction syndrome [103–105].

Prophylactic treatment to avoid abdominal compartment syndrome involves refraining from abdominal closure when fascial approximation becomes problematic due to excessive tension (“open abdomen”) [106, 108]. In this setting, negative pressure peritoneal therapy may play a role in mitigating the bio-mediator effects that cause distant organ failure and is an additional potential benefit of an open abdomen.

Even in cases where the abdominal wall can be closed after a laparotomy involving the discovery of diffuse contamination, fulfilling the World Society of Emergency Surgery criteria for severe complicated intra-abdominal sepsis [107, 108], there is controversy as to whether the abdominal wall should be closed or left open. It is financially cheaper and would be preferable from a patient’s standpoint to have a single operation and to not be submitted to longer critical care unit management if it was possible to primarily close the abdomen [109]. However, there is a now developing biologic rationale with early clinical evidence that the open abdomen after severe complicated intra-abdominal sepsis may be preferable due to its ability to allow negative pressure peritoneal therapy which may modulate the course of systemic inflammation with progressive organ dysfunction [110, 111] and to provide a survival signal that needs to be confirmed in larger studies [112, 113].

Following stabilization of the patient, surgeons should attempt early, definitive closure of the abdomen. Primary fascial closure may be possible only when the risk of excessive tension or recurrent IAH is minimal (grade 2C recommendation).

When early definitive fascial closure is not possible, progressive closure can be gradually attempted at every surgical wound revision. Cross-linked biological meshes may be considered as a delayed option for abdominal wall reconstruction (grade 2C recommendation).

After the patient’s stabilization, the primary objective is early and definitive closure of the abdomen to minimize complications. For many patients, primary fascial closure may be possible within a few days of the first operation. In other patients, early definitive fascial closure may not be possible. In these cases, surgeons must resort to progressive closure, in which the abdomen is incrementally closed each time the patient undergoes a surgical revision. Many methods of fascial closure have been described in the medical literature [94, 114–117].

In 2012, a retrospective analysis evaluating the use of vacuum-assisted closure and mesh-mediated fascial traction (VACM) as temporary abdominal closure was published. The study compared 50 patients treated with VACM and 54 using non-traction techniques (control group). VACM resulted in a higher fascial closure rate and lower planned hernia rate than methods that did not provide fascial traction [117].

Occasionally, abdominal closure is only partially achieved, resulting in large, debilitating hernias of the abdominal wall that will eventually require complex surgical repair. Bridging meshes will often result in bulging or recurrences [118]. The Italian Biological Prosthesis Working Group (IBPWG) proposed a decisional algorithm in using biological meshes to restore abdominal wall defects [64].

When definitive fascial closure cannot be achieved, a skin-only closure is a viable option and subsequent eventration can be managed at a later stage with delayed abdominal closure and synthetic mesh repair (grade 1C recommendation).

Damage control surgery has been widely used in trauma patients, and its use is rapidly expanding in the setting of acute care surgery. Damage control surgery can be used in patients with strangulated obstruction and peritonitis caused by bowel perforation with enteric spillage due to a complicated abdominal wall hernia. These patients are often considered critically ill due to septic complications. Ordonez et al. described a series of 217 non-trauma patients with severe peritonitis and who were managed with damage control surgery. Definitive fascia closure was achieved in 51% of the patients. Failure of definitive fascia closure occurred in 106 patients; of these, 72 (68%) were managed with skin-only closure. Skin-only closure could be an alternative for patients with failure of definitive fascia closure, reducing the risk of complications of open abdomen and abdominal compartmental syndrome. Patients could be deferred for delayed definitive abdominal closure with synthetic mesh repair [119].

The component separation technique may be a useful and low-cost option for the repair of large midline abdominal wall hernias (grade 1B recommendation).

The component separation technique (CST) for reconstructing abdominal wall defects without the use of prosthetic material was described in 1990 by Ramirez et al. [120]. The technique is based on enlargement of the abdominal wall surface by translation of the muscular layers without damaging the muscle innervation and blood supply [121]. In most series, several modifications to the original technique have been performed, including the use of prosthetic material [122–125]. In a prospective randomized trial comparing CST with bridging the defect with a prosthetic material, CST was found to be superior, although a similar recurrence rate was found after a 24-month follow-up [126]. However, high recurrence rates (up to 38.7%) after component separation have recently been reported [127].

The microvascular tensor fasciae latae (TFL) flap is a feasible option for reconstruction of exceptionally large abdominal wall defects. This technique can also be combined with other methods of reconstruction. Vascularized flaps provide healthy autologous tissue coverage without implantation of foreign material at the closure site. A close collaboration between plastic and abdominal surgeons is important for this reconstruction [128].

### Antimicrobial prophylaxis

In patients with intestinal incarceration with no evidence of ischaemia and no bowel resection (CDC wound class I), short-term prophylaxis is recommended (grade 2C recommendation).

In patients with intestinal strangulation and/or concurrent bowel resection (CDC wound classes II and III), 48-h antimicrobial prophylaxis is recommended (grade 2C recommendation).

Antimicrobial therapy is recommended for patients with peritonitis (CDC wound class IV, grade 2C recommendation).

In aseptic hernia repair, *Staphylococcus aureus* from the exogenous environment or the patient’s skin flora is typically the source of infection. In patients with intestinal strangulation, the surgical field may be contaminated by bacterial translocation [8, 9] from intestinal villi of incarcerated ischemic bowel loops as well as by concomitant bowel resections. In patients with peritonitis, both antimicrobial therapy and surgery are always recommended.

### Anaesthesia

Local anaesthesia (LA) can be used, providing effective anaesthesia with less postoperative complications for emergency inguinal hernia repair in the absence of bowel gangrene (grade 1C recommendation).

LA is one of the most commonly used anaesthetic methods in inguinal hernia repair [129–131]. However, the role of LA in emergency inguinal hernia repair is still controversial [132–134]. The recent retrospective 5-year experience by Chen et al. reported that LA could provide effective anaesthesia and patient safety in emergency inguinal hernia repair, with less cardiac complications (*P* = 0.044) and respiratory complications (*P* = 0.027), shorter ICU stay (*P* = 0.035) and hospital stay (*P* = 0.001), as well as lower cost (*P* = 0.000) and faster recovery time (*P* = 0.000) than general anaesthesia [135].

However, general anaesthesia should be preferred in the case of suspected bowel gangrene and need of intestinal resection and always in the case of peritonitis.

## Conclusions

Emergency repair of complicated abdominal hernias remains one of the most common and challenging surgical emergencies and is associated with a significant burden for health care systems worldwide. These comprehensive guidelines on the emergency repair of complicated hernia have been developed by a panel of experts through a Web-based discussion and consensus. This document provides evidence-based recommendations on the timing of intervention, laparoscopic approach, surgical repair according to the CDC wound classification, and antimicrobial prophylaxis on the topic of emergency repair of complicated abdominal wall hernias. One of the novel aspects of the present guidelines is the stratification of the management recommendations according to the CDC wound classification, which is a widely used and standardized classification of the surgical wounds. In addition, this 2017 revision includes a new topic on the role of local anaesthesia.

## Appendix

**Table 3:** Resume of recommendation guidelines

GoR	Recommendation
Timing of intervention	
1C	Patients should undergo emergency hernia repair immediately when intestinal strangulation is suspected
1C	Systemic inflammatory response syndrome (SIRS), contrast-enhanced CT findings, as well as lactate, CPK, and D-dimer levels are predictive of bowel strangulation
Laparoscopic approach	
2B	Diagnostic laparoscopy may be a useful tool with the target of assessing bowel viability after spontaneous reduction of strangulated groin hernias
2C	Repair of incarcerated hernias—both ventral and groin—may be performed with a laparoscopic approach in the absence of strangulation and suspicion of the need of bowel resection, where an open preperitoneal approach is preferable
Emergency hernia repair in “clean surgical field” (CDC wound class I)	
1A	The use of mesh in clean surgical fields (CDC wound class I) is associated with a lower recurrence rate, if compared to tissue repair, without an increase in the wound infection rate. Prosthetic repair with a synthetic mesh is recommended for patients with intestinal incarceration and no signs of intestinal strangulation or concurrent bowel resection (clean surgical field)
Emergency hernia repair in “clean-contaminated surgical field” (CDC wound class II)	
1A	For patients having complicated hernia with intestinal strangulation and/or concomitant need of bowel resection without gross enteric spillage (clean-contaminated surgical field, CDC wound class II), emergent prosthetic repair with synthetic mesh can be performed (without any increase in 30-day wound-related morbidity) and is associated with a significant lower risk of recurrence, regardless of the size of hernia defect
Emergency hernia repair in “contaminated-dirty surgical field” (CDC wound classes III and IV)	
2C	For stable patients with strangulated hernia with bowel necrosis and/or gross enteric spillage during intestinal resection (contaminated, CDC wound class III) or peritonitis from bowel perforation (dirty surgical field, CDC wound class IV), primary repair is recommended when the size of the defect is small (< 3 cm); when direct suture is not feasible, a biological mesh may be used for repair
2C	The choice between a cross-linked and a non-cross-linked biological mesh should be evaluated depending on the defect size and degree of contamination
2C	If biological mesh is not available, either polyglactin mesh repair or open wound management with delayed repair may be a viable alternative
2C	For unstable patients (experiencing severe sepsis or septic shock), open management is recommended to prevent abdominal compartment syndrome; intra-abdominal pressure may be measured intraoperatively
2C	Following stabilization of the patient, surgeons should attempt early, definitive closure of the abdomen. Primary fascial closure may be possible only when the risk of excessive tension or recurrent intra-abdominal hypertension (IAH) is minimal
2C	When early definitive fascial closure is not possible, progressive closure can be gradually attempted at every surgical wound revision. Cross-linked biological meshes may be considered as a delayed option for abdominal wall reconstruction
1C	When definitive fascial closure cannot be achieved, a skin-only closure is a viable option and subsequent eventration can be managed at a later stage with delayed abdominal closure and synthetic mesh repair
1B	The component separation technique may be a useful and low-cost option for the repair of large midline abdominal wall hernias
Antimicrobial prophylaxis	
2C	In patients with intestinal incarceration with no evidence of ischaemia and no bowel resection (CDC wound class I), short-term prophylaxis is recommended
2C	In patients with intestinal strangulation and/or concurrent bowel resection (CDC wound classes II and III), 48-h antimicrobial prophylaxis is recommended
2C	Antimicrobial therapy is recommended for patients with peritonitis (CDC wound class IV)
Anaesthesia	
1C	LA can be used, providing effective anaesthesia with less postoperative complications for emergency inguinal hernia repair in the absence of bowel gangrene

## Abbreviations

CDC: Centers for Disease Control and Prevention
OR: odds ratio
RCT: randomized controlled trial
WSES: World Society of Emergency Surgery
